# An *In Situ* Temperature-Dependent Study of La_2_O_3_ Reactivation Process

**DOI:** 10.3389/fchem.2021.694559

**Published:** 2021-05-31

**Authors:** Xiaohong Zhou, Evgeny I. Vovk, Yang Liu, Cairu Guan, Yong Yang

**Affiliations:** ^1^ School of Physical Science and Technology, ShanghaiTech University, Shanghai, China; ^2^ Shanghai Institute of Optics and Fine Mechanics, Chinese Academy of Sciences, Shanghai, China; ^3^ Shanghai Institute of Optics and Fine Mechanics, University of Chinese Academy of Sciences, Beijing, China

**Keywords:** *in situ* XPS, *in situ* XRD, online MS, La_2_O_3_, reactivation

## Abstract

Lanthanum-containing materials are widely used in oxidative catalytic and electrocatalytic reactions such as oxidative coupling of methane (OCM) and solid oxide fuel cells (SOFCs). However, many of these materials are highly susceptible to air contamination which means *ex situ* characterization results generally cannot be associated with their reactivity. In this study, the activation processes of an *in situ*–prepared bulk La_2_O_2_CO_3_ sample and an *ex situ* as-prepared La(OH)_3_ sample are *in situ* investigated by X-ray photoelectron spectroscopy (XPS), X-ray diffraction (XRD), and online mass spectroscopy (MS). Results indicate that the La_2_O_2_CO_3_ sample, during linear heating to 800°C, always contains some carbonates near the surface region, which supports a two-step model of bulk carbonate decomposition through surface sites. The La(OH)_3_ sample structure evolution is more complex due to contaminations from air exposure. Together with TGA results, online mass analysis of water and CO_2_ signal loss showed that three major catalyst structure phase change steps and a preheating up to 800°C are required for the as-prepared material to be transferred to La_2_O_3_. This process is carefully investigated combining the three *in situ* methodologies. XPS and XRD data further reveal transformations of variety of *in situ* surface structures and forms including hybrid phases with hydroxyl, carbonates, and oxide as the sample heated to different temperatures within the range from 200 to 800°C. The results provide useful insights on the activation and deactivation of La-contained materials.

## Introduction

The rare earth lanthanum is contained in a wide and important class of oxidation catalysts and electrocatalysts such as perovskites and MOF (Alcalde-Santiago et al., 2020; Lee, 2020; Karuppiah et al., 2021). These materials can be used as deep oxidation catalysts for a wide range of environmental applications (Hu et al., 2021; Ishisone et al., 2021; Le et al., 2021), which include membrane materials, electrocatalytic materials (Brugge et al., 2020), and materials in solid oxide fuel cells (SOFCs) (Duan et al., 2020). La_2_O_3_ is also widely used in materials applications, such as optical glasses, light-emitting materials, laser materials, and hydrogen storage materials (Dong et al., 2020; Le et al., 2021). On the other hand, many materials development adapt modification with La as it may help tailoring electronic, thermodynamic, and catalytic properties. Both the surface and bulk properties could be affected by these compositional changes. For most of these materials, La compounds are found on the surface (Vovk et al., 2005; Brugge et al., 2020; Duan et al., 2020; Sun et al., 2020; Hu et al., 2021; Ishisone et al., 2021; Le et al., 2021). Many of surface species of this group of materials are not stable under the operation conditions (Sunding et al., 2011; Li et al., 2019). In addition, after air exposure, their surface structure under operation conditions is usually not preserved. After the samples being transferred into ultrahigh vacuum (UHV) environment for traditional surface analysis, an *in situ* sample reactivation is required (Dou et al., 2017; Dong and Vayssieres, 2018; Nguyen et al., 2019; Zhang et al., 2019; Han et al., 2021). However, little is known about how the lanthanum-containing surface structure changes upon oxidation and reduction, including the status of surface oxygen species and other add-on species such as carbonates and adventitious carbon.

One of the most important applications of La_2_O_3_ as a catalyst is oxidative coupling of methane (OCM), in which La_2_O_3_-based catalysts exhibit excellent catalytic performance and give a commercialization potential (DeBoy and Hicks, 1988; Huang et al., 2013; Song et al., 2015; Jiang et al., 2016; Aseem et al., 2018; Gambo et al., 2018; Sollier et al., 2018). OCM reaction produces ethylene, a vital building block in the chemical industry (Gao et al., 2019), by direct coupling of CH_3_ radicals in the gas phase. It is considered as one of the promising ethylene production processes (Galadima and Muraza, 2016; Taifan and Baltrusaitis, 2016). Previous publications (Le Van, 1993; Lacombe et al., 1995; Xu et al., 1995), including our recent *in situ* studies, indicated that La_2_O_3_ is not stable in this reaction (Liu et al., 2018; Li et al., 2019; Guan et al., 2021; Pang et al., 2021; Zhou et al., 2021). In our recent studies, it was strongly suggested that a high temperature (800°C) precleaned sample surface, which is free of carbonates and hydroxyls, is enriched with a specific subsurface peroxide species (Zhou et al., 2021). A higher selectivity of C_2_ products was found on this surface. However, with high concentration (usually >10%) of CO_x_ as the reaction byproducts, the La_2_O_3_ sample is generally found forming, at least partially, surface and bulk La_2_O_2_CO_3_ (Le Van, 1993). With such conditions, the reaction selectivity and activity are found to be reduced (Xu et al., 1995; Guan et al., 2021). In addition, hydroxyl is also found to be easily formed (Li et al., 2019).

Our previous *in situ* XPS studies, applying a UHV-connected high pressure gas cell (HPGC), investigated pure La_2_O_3_ sample pretreated by water, CO_2_, O_2_, H_2_, CH_4_, and OCM reactants at different temperatures (Li et al., 2019). The results provide a reliable procedure for XPS binding energy (BE) scale calibration by applying carbon (deposited by *in situ* methane treatment) and lattice oxygen peaks as internal benchmarks. Results also showed that the binding energy of La 4d_7/2_ is the easiest benchmark for XPS spectra calibration as its BE is always uniform (102.2 ev) after above pretreatments including air exposure. A recent DFT simulation further corroborated the experimental results revealing the catalyst surface structure under realistic process conditions (Pang et al., 2021). Using the pristine La_2_O_3_ bulk structure supercell as reference, results suggested more proper definition of previous XPS feature assignments. For example, DFT found that the binding energy of the 4-coordinator (O4c) oxygen site in the (La_2_O_2_^2+^)_n_ layer of bulk La_2_O_2_CO_3_ overlaps with the lattice oxygen in La_2_O_3_.

In this study, two samples, that is, La_2_O_2_CO_3_ (*in situ*–prepared) and La(OH)_3_ (*ex situ*–prepared), are investigated by *in situ* XPS, XRD, and online MS for their surface and bulk structures. As these two samples represent two most popular La-related contamination species on many complex oxide surfaces, such as perovskites and MOF, their temperature stability and evolution of structure and phases are observed in detail. The *in situ* measurements are continued until the complete regeneration of La_2_O_3_ at about 800°C. All observed species are further interpreted by the characteristic species assignments and DFT surface structure model mentioned above. This study hopefully provides useful insight for understanding the related behavior of La-containing materials and the reaction performance of La-based catalysts.

## Materials and Methods

### Sample Preparation

The fresh nanorod La_2_O_3_ catalyst used in this work is described in detail elsewhere. The synthesis of the La_2_O_3_ nanorod catalysts follows the process proposed by Zhu and coworkers (Huang et al., 2013). In this study, a sample after this synthesis preparation is referred as “fresh as-prepared sample” which is mostly composed with La(OH)_3_. The SEM result indicates the sample is in the shape of nanorods with 10 nm diameter in average. BET measurement (N_2_) yields specific surface area of 3.4 m^2^/g (Liu et al., 2018).

Previous XPS and XRD analysis of the fresh as-prepared nanorod sample, after heating in vacuum at the temperature of 800°C for 1 h and subsequent cooling to room temperature at the same conditions, demonstrate a pure lanthanum oxide with minimum hydroxide and carbonate in the bulk or on the surface (Li et al., 2019; Guan et al., 2021; Zhou et al., 2021). In this work, the sample after this preheating in vacuum procedure is referred as “precleaned sample.”

The precleaned sample, after exposing to pure CO_2_ for 1 h at 600°C in HPGC and cooling down in the same atmosphere, is completely transferred into La_2_O_2_CO_3_. In this study, it is referred as “*in situ*–prepared La_2_O_2_CO_3_.”

### Characterization Techniques

#### *In Situ* XPS

The XPS surface analysis in this work is performed by the ThermoFischer ESCALAB 250Xi XPS spectrometer. All spectra were acquired using a monochromated X-ray irradiation Al*K*α (*hv* = 1486.7 eV, 300 W, 500 μm spot size) and a 180° double-focusing hemispherical analyzer with a six-channel detector (30 eV pass energy). For *in situ* heating experiments simulating industrial high-temperature pretreatments, the sample analysis chamber (SAC) was specially equipped with a homemade heating stage. This sample stage is designed to be compatible with the original spectrometer manipulator which allows a sample to be heated up to 800°C with simultaneous XPS analysis. The *in situ* heating stage is also adapted to the regular flag-sample holder which allows the sample to be transferred to all the other UHV-connected functional chambers within the vacuum system. All heating treatments, if not specified additionally, are performed in the analysis chamber.

For XPS analysis, the fresh powder sample was pressed in a tiny 1.5 mm hole of a flag-shaped sample holder and then preheated in vacuum (10^−10^mbar). The *in situ* XPS analysis for every step is conducted at the fixed temperature. The analysis location (Φ=500 μm ) on the sample surface is chosen randomly. A set of primary sample spectra (C 1s, O 1s, La 3d, and La 4d) were acquired at 25°C before heating starts. Similarly, spectra were collected between 200 and 800°C with an increment of 100°C. Finally, the spectra after subsequent cooling to the room temperature were also collected. The monitoring time for spectra collected at fixed temperature was around 10°min; the heating rate in the XPS experiments was 10°C/min. During the whole process, the maximum pressure in the analysis chamber monitored by a wide-range gauge was around 2·10^−8^ mbar. All spectra processing analysis including O 1s and C 1s peak fitting are performed in Thermo Avantage software. The spectra are deconvoluted using the Lorentz–Gauss mix function after subtraction of modified Shirley (Smart) background. As proposed in our previous work (Li et al., 2019), the binding energy scale of all photoelectron spectra is calibrated to La 4d_5/2_ main peak at 102.2 eV. The atomic ratios of surface species are calculated from the corresponding photoelectron peaks after background subtraction taking into account transmission function and atomic sensitivity factors (ASF).

#### *In Situ* XRD

To confirm the phase change during the first heating of a fresh La_2_O_3_ sample, an *in situ* powder X-ray diffraction (*in situ* XRD) was used. *In situ* XRD analysis was conducted in the Bruker D8 Advance X-ray diffraction system using *in situ* Anton Paar XRK 900 cell reactor. The system is equipped with Cu Kα X-ray source (*λ* = 0.154 nm, 20 kV, 20 mA). The diffraction patterns are acquired with a step width of 0.02° in the (2θ) range from 20 to 50°. The sample temperature was ramped from room temperature to 800°C at a rate of 10°C/min. All presented survey scans are obtained at specified temperature.

#### Online MS

Temperature program desorption (TPD) in vacuum for a fresh sample was carried out using a quadrupole mass spectrometer (QMS, SRS 300) connected to HPGC (Zhou et al., 2021).

## Results and Discussion

### Activation of *In Situ*–Prepared La_2_O_2_CO_3_

**FIGURE 1:**
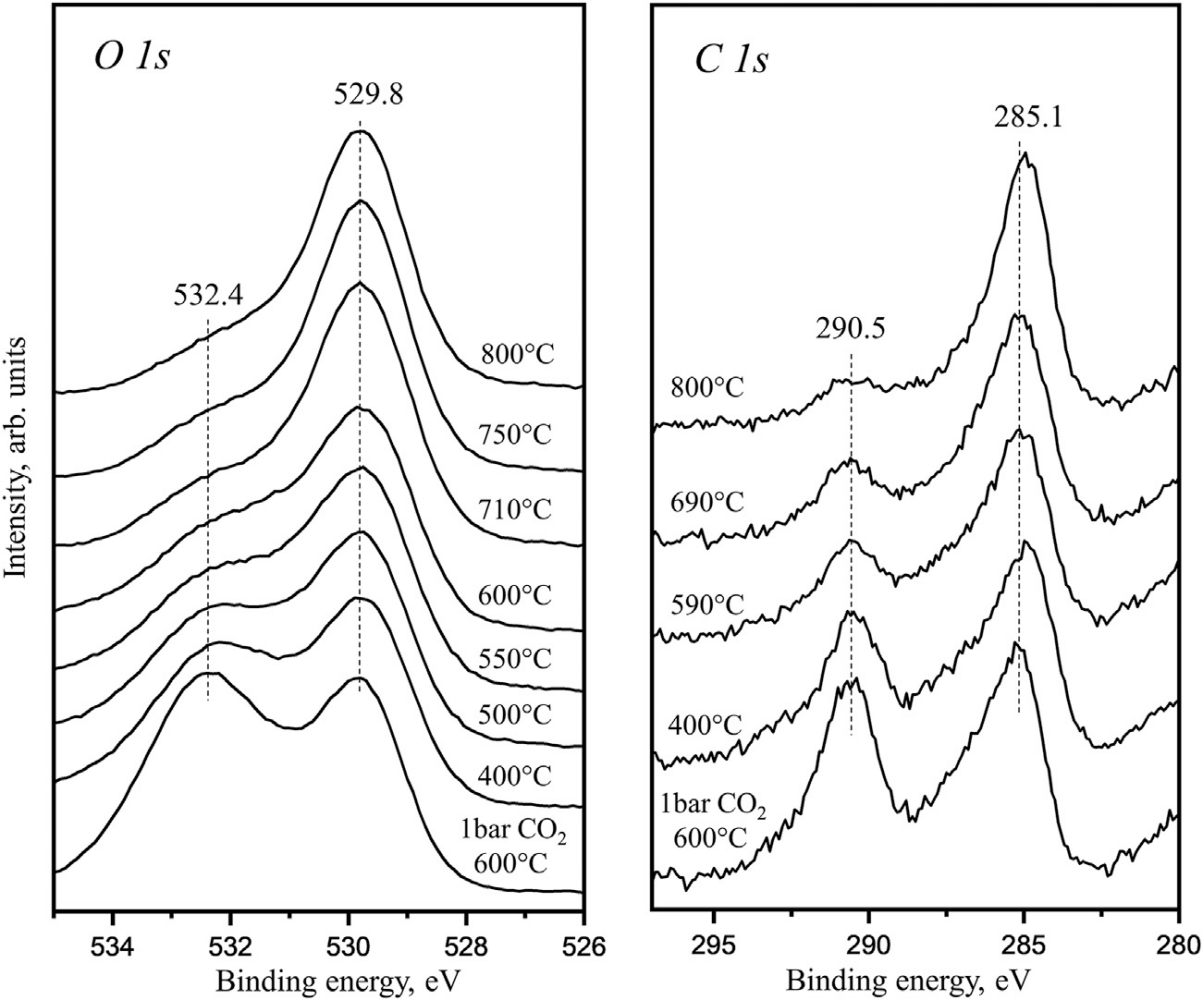
*In situ* XPS C 1s and O 1s core level spectra obtained in the temperature range from 400 to 800°C after pretreatment under 1 bar CO_2_ in UHV-connected HPGC.

Figure 1 data are obtained as the *in situ*–prepared La_2_O_2_CO_3_ is linearly heated directly in front of analyzer during the XPS analysis. The presented C 1s and O 1s spectra are collected at the actual temperature. The 529.8 eV peak in O 1s spectra is related to lattice oxygen in La_2_O_3_ or 4-coordinated oxygen atoms in La_2_O_2_CO_3_. The C 1s peak with BE around 285 eV is associated with adventitious carbon accumulated on the sample surface from background during the heating in HPGC. Both C 1s and O 1s spectra also show characteristic peaks of CO_3_^2−^ groups (BEs 532.4 eV for O 1s and 290.5 eV for C 1s). Our previous TPD studies showed that the bulk La_2_O_2_CO_3_ demonstrates two major desorption features: decomposition of the surface carbonate below 550°C and decomposition of the bulk La_2_O_2_CO_3_ from 600 to 800°C (Liu et al., 2018). During this sample linear heating to 800°C, the photoelectron spectra show that at 600°C (surface carbonate decomposition), the intensity of the carbonate species is strongly reduced. When the temperature reaches 800°C, a small carbonate signal in C 1s region is still observed. This species cannot be related to the initial surface carbonates but should be assigned to the bulk species decomposition through the surface. Therefore, the XPS results combined with the previous TPD data suggest that the bulk carbonate decomposes through a two-step process: the 1^st^ step is migration to the surface and the 2^nd^ is surface desorption. This is in agreement with our previous conclusion from kinetics study and DFT simulation results of bulk and surface carbonate free energies (Liu et al., 2018; Zhou et al., 2021). This *in situ* observed surface electronic structure also clearly indicates that during the linear heating, the continuous decomposition of bulk La_2_O_2_CO_3_ will result in surface sites occupied by carbonate groups at high temperature. In the La_2_O_3_-catalyzed OCM reaction, such surface carbonate occupation has a poisoning effect (Guan et al., 2021).

### Activation of La(OH)_3_ (Fresh As-Prepared Sample)

#### TGA and Online MS Analysis

**FIGURE 2:**
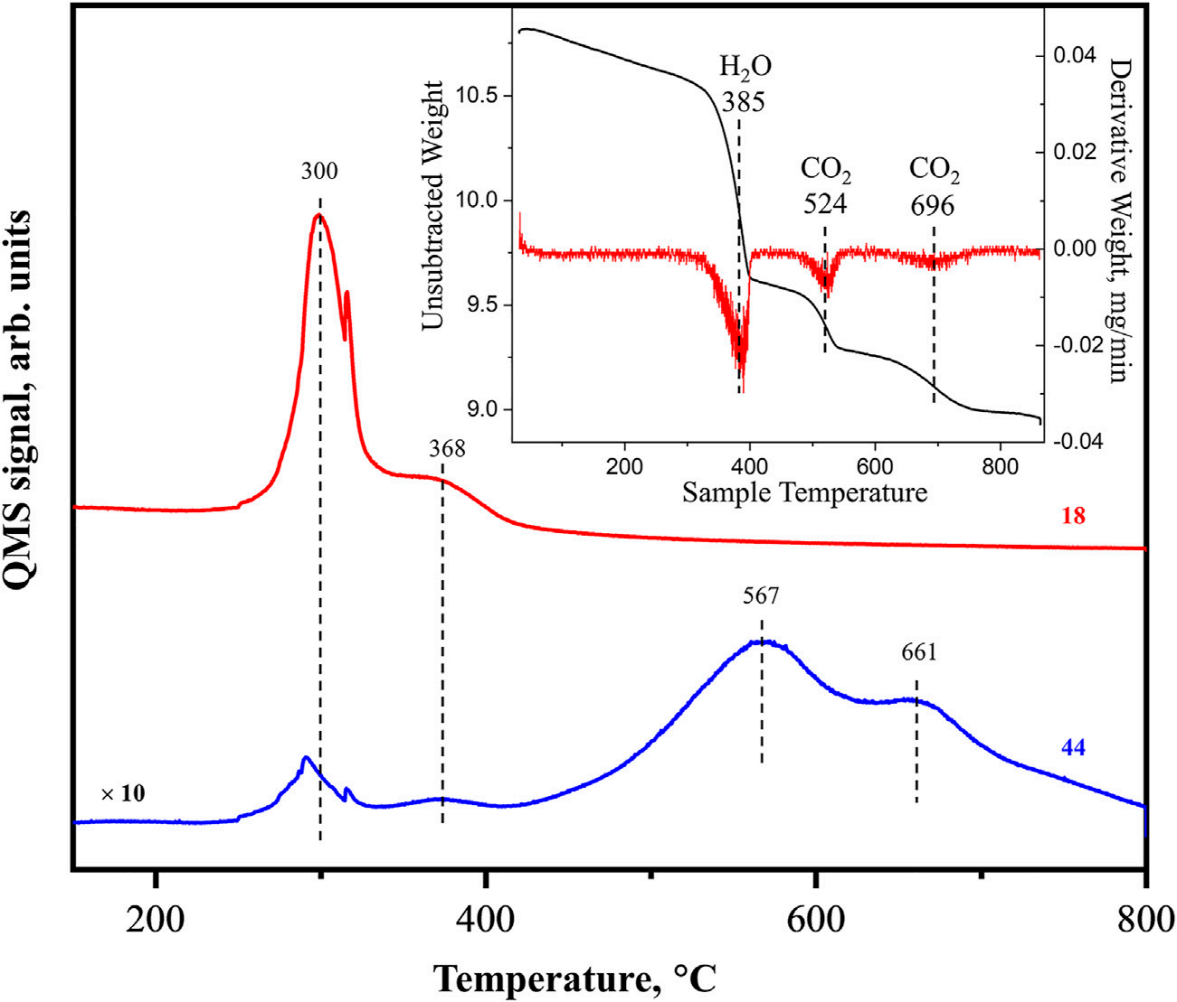
MS data of as-prepared La_2_O_3_ sample heated from room temperature to 800°C, showing the desorption signal of H_2_O (amu = 18, red) and CO_2_ (amu = 44). Inset: Thermogravimetric analysis (TGA) weight loss curve of La_2_O_3_ sample under N_2_ flow (black) and derivative (red) from Li et al. (2019).

To *in situ* obtain pure La_2_O_3_, the fresh nanorod sample after the synthesis needs to be heated to 800°C in inert gas or oxygen to decompose hydroxide and carbonate (Liu et al., 2018; Li et al., 2019; Guan et al., 2021). Figure 2 shows the online MS data of water (amu 18) and CO_2_ (amu 44) desorbing from the freshly prepared sample during the heating process. The inset of the figure plots our previously obtained TGA result including the raw data (black curve) and its derivative curve (red) for a comparison (Li et al., 2019). The combined results show that the process consists of 3 steps of mass change. In the 1st step at around 380°C, about 8.5% loss of total mass is observed which is identified as H_2_O desorption within a narrow 50°C temperature range. A small CO_2_ desorption signal (∼2% of water) is also detected at this step. No more water signal is observed in later TPD process, and this step is considered as sample dehydration. In the 2nd step at about 530°C, around 2.5% loss of total mass is observed which is identified as partial carbonate decomposition since it is accompanied by CO_2_ desorption. Finally, within 3rd step at around 700°C, about 3.0% mass loss is observed and identified as the complete carbonate decomposition accompanied by CO_2_ desorption (Li et al., 2019). The MS signal indicates the two CO_2_ desorption peaks which are much broader than the water related one. The higher temperature CO_2_ desorption peak is less intense than the lower temperature peak manifesting itself as a shoulder. After heating to 800°C, TGA demonstrates the total sample mass leftover is 9.0 mg. As mentioned in the introduction section, the sample after *in situ* heating up to this temperature is pure La_2_O_3_. Applying the recorded mass loss and the knowing molar masses, the molar ratio of H_2_O:CO_2_:La_2_O_3_ is roughly 3.4:1:2. In literature (Hou et al., 2015), similar three steps of mass loss were reported before. The transformations during the heating were explained as follows: in the 1st step, a mixture of La_2_(OH)_4_CO_3_ and La(OH)_3_ phases are decomposed forming La_2_O_2_CO_3_ and LaOOH, respectively, both yielding H_2_O as desorbed product. During the 2nd and 3rd steps, La_2_O_2_CO_3_ is decomposed to La_2_O_3_ yielding CO_2_ in two stages at ∼ 520 and 700°C.

#### *In Situ* Heating From Room Temperature to 400°C (Sample Dehydration)

Supplementary Figure S1 presents C 1s and O 1s photoelectron spectra of an as-prepared fresh sample and after *in situ* precleaning procedure by heating in vacuum at 800°C for 1 h. The C 1s spectrum of the as-prepared sample shows two main peaks with similar intensities: the adventitious carbon peak at 284.6 eV and a higher binding energy peak at 289.5 eV associated with carbonate. The O 1s spectrum of the as-prepared fresh sample has three features: a main peak at 531.0 eV and two relatively small shoulders at 532.3 and 528.7 eV. The shape of C 1s and O 1s spectra correlate very well with our previously published data (Li et al., 2019). After *in situ* precleaning procedure by heating in vacuum, both carbon features are removed. The O 1s spectrum after *in situ* cleaning shows one main peak at 529.8 eV assigned to lattice oxygen in La_2_O_3_ and a broad shoulder at 531.6 eV assigned to La peroxide (Zhou et al., 2021).

The above O 1s and C 1s peaks on the as-prepared surface are related to carbonates, adventitious carbon, and lattice oxygen. However, BE of these peaks are all somehow different from the peaks obtained for the *in situ* cleaned surface, although they are all calibrated with the La 4d peak as the standard procedure in our previous work (Li et al., 2019). This indicates that the actual species composition of the as-prepared surface could be more complex than the adsorbates *in situ* formed on the precleaned La_2_O_3_. To *in situ* investigate the surface electronic structure evolution of the catalyst during the sample precleaning process, the C 1s and O 1s core level spectra of the as-prepared fresh sample are obtained during simultaneous step heating directly in SAC of the XPS spectrometer. In addition, the similar heating process is also conducted in the *in situ* XRD reactor with the same heating profile. Comparing the two sets of data, the substantial bulk and surface electronic structure changes before and after the heating procedure are considered in multiple gradually changing steps. The results are presented following these steps of heating.

**FIGURE 3:**
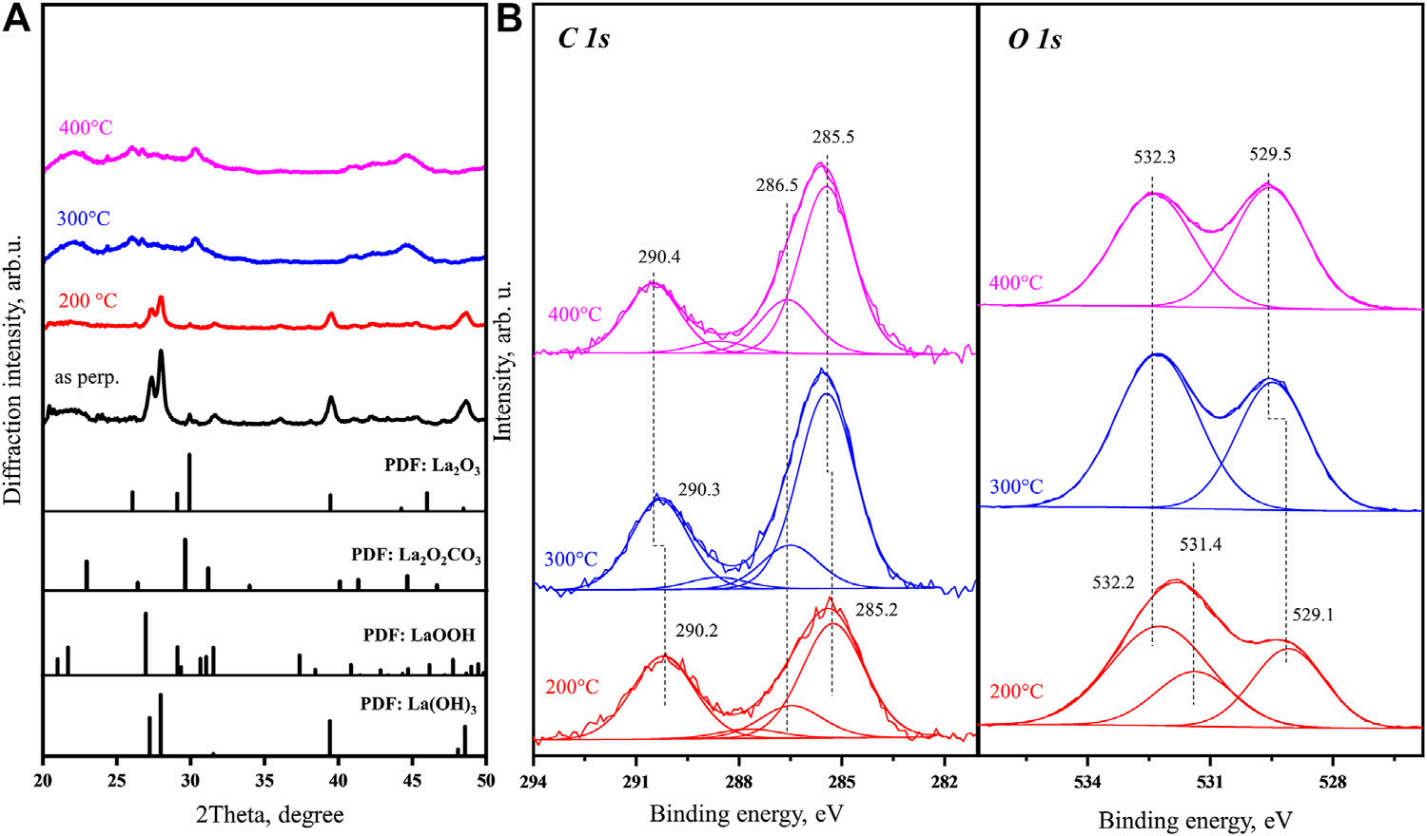
**(A)** *In situ* XRD patterns obtained for as-prepared fresh La_2_O_3_ sample at room temperature, 200, 300, and 400°C with PDF database of La(OH)_3_, La_2_O_2_CO_3_, LaOOH, and La_2_O_3_ as reference. **(B)** *In situ* C 1s and O 1s core level XPS spectra obtained for an as-prepared fresh nanorod La_2_O_3_ sample held at 200, 300, and 400°C.

Figure 3A shows four *in situ* XRD patterns of the as-prepared fresh sample obtained at room temperature, 200, 300, and 400°C in comparison to PDF reference data for standard La compounds. At room temperature and 200°C, the sample bulk structure is dominated by La(OH)_3_. After the sample is heated to 300°C, the XRD pattern is significantly changed. Comparing with known PDF database, the main peaks in La(OH)_3_ diffraction pattern are not observed in the new pattern, suggesting that the La(OH)_3_ is completely decomposed. TGA and MS results above also support that heating to 300°C is accompanied by simultaneously removing most of hydroxide species from the bulk. Therefore, the main contamination in the sample bulk should be carbonates. However, the peaks in the new XRD patterns obtained at 300 and 400°C [attributed mainly to tetragonal La_2_O_2_CO_3_ (011)] are not found in good consistency with any single XRD PDF of the known La-based compounds. In addition, all the peaks obtained, such as the one with the highest intensity at 30.3°, are very broad, which indicates that after heating to 300°C, the new crystallization is only started forming very small grains. Most of the peaks in the obtained experimental XRD patterns can be covered by superposition of the tetragonal La_2_O_2_CO_3_ and hexagonal La_2_O_3_ PDF data. The behavior of XRD pattern suggests that the carbonate is highly dispersed in the sample below 300°C. However, there are still a few broad peaks in the experimental curves which are different from the carbonate and oxide references. This indicates that the sample, after being exposed to air and contaminated, is still not only a mixture of La(OH)_3_ and La_2_O_2_CO_3_ but also contains other complex compounds, such as La_2_(OH)_4_CO_3_ or LaOOH.

The *in situ* XP spectra obtained for the same batch of sample are shown in Figure 3B. As the sample is *in situ* heated up to 200°C, the C 1s peak shape is not changed significantly. The O 1s spectrum taken at 200°C shows the 529.1 eV peak which can be assigned to La_2_O_3_ lattice oxygen (Sunding et al., 2011) and a broad higher BE peak which can be deconvoluted into two features at 531.4 and 532.2 eV. We associate these peaks with surface hydroxide and carbonate, respectively, taking into account our previous data for lanthanum oxide after interaction with H_2_O and CO_2_ (Li et al., 2019). At 300 and 400°C, the observed spectra are already in good agreement (less than 0.3 eV in peaks BE difference) with our previously published calibrated results for carbonated surface (Li et al., 2019). The O 1s and C 1s spectra show clear carbonate peaks with binding energies 532.5 and 290.5 eV. 532.5 eV is typical position of the oxygen in the CO_3_^2-^ group, either carbonate on La_2_O_3_ surface or bulk La_2_O_3_CO_3_ (Li et al., 2019; Pang et al., 2021). The O 1s spectrum also shows a sharp oxygen peak with binding energy of 529.5 eV. As mentioned before, 529.5 eV peak is generally assigned to lattice oxygen in La_2_O_3_. Our recent study demonstrated that it can also be associated with 4-coordinated oxygen (O4c) in (La_2_O_2_^2+^)_n_ layer of bulk La_2_O_2_CO_3_ (Pang et al., 2021). No hydroxyl peak in O 1s spectra is observed at 300 and 400°C as the TGA and MS data also indicate that the sample dehydration at this temperature is nearly complete. These XPS spectra support that heating of the as-prepared fresh sample to 400°C results in nearly complete dehydration with carbonate species on the surface starting to show up.

Comparison of the *in situ* data provides a more comprehensive assignment of the 528.7 eV peak observed in the O 1s spectrum of the as-prepared fresh sample (Supplementray Figure S1). An earlier publication assigned this low binding energy shoulder to lattice oxygen of La_2_O_3_ (Baškys et al., 2014; Jiang et al., 2016); other researchers attribute this peak to oxygen atoms formed after partial dehydration of La(OH)_3_ in vacuum or due to X-ray irradiation (Sunding et al., 2011). In above heating from room temperature to 400°C, the initial 528.7 eV O 1s peak observed in our *in situ* experiments is clearly approaching the BE of 529.8 eV. Our previous results revealed that the O 1s binding energy of hydroxyl is exclusively around 531.8 eV, which cannot be associated with either 529.5 eV or the 528.7 eV (Li et al., 2019). On the other hand, *in situ* XRD results indicate that there is no La_2_O_3_ phase at 200°C. The combined TGA and XRD results also support that the sample is changed from a mixture of La(OH)_3_ and La_2_O_2_CO_3_ at 200°C to a mixture of La_2_O_2_CO_3_ and La_2_O_3_ at 400°C during a gradual dehydration. For the O 1s peak with binding energy at ∼ 529.5 eV observed at 400°C, our previous publication assigned it to either the characteristic lattice oxygen species [(O4c) and (O6c)] in La_2_O_3_ or the (O4c) in the (La_2_O_2_^2+^)_n_ layer of bulk La_2_O_2_CO_3_ (Pang et al., 2021). Figure 3 shows that this peak could be as low as 528.7 eV on the as-prepared fresh sample with heavy concentration of hydroxyl species. As the temperature approaching 400°C, this peak moves to 529.5 eV. On the other hand, the behaviors of other O 1s and C 1s features show similar shifting during the dehydration procedure. At 200°C, the dominant peak in O 1s spectrum of the as-prepared fresh sample with binding energy 531.0 eV also slightly shifts to higher value of 531.5 eV, which is the characteristic peak position for hydroxyl. In reverse to the other peaks, the intensity of the hydroxyl peak, during its shifting process, is continuously decreasing. This is in agreement with the online MS result of water loss in this temperature range. In the C 1s spectra, the original 289.5 eV peak of the as-prepared fresh sample shifts gradually to 290.2 eV (200°C), 290.3 eV (300°C), and 290.4 eV (400°C), approaching the characteristic binding energy of carbonates (290.5 eV). Noticing that along with dehydration in UHV, the carbonate peak intensity is continuously increasing; this indicates that the as-prepared fresh sample surface is enriched with hydroxyl. Overall, the O 1s and C 1s spectra obtained below 300°C do not have typical features fully consistent with carbonates, hydroxyl, or La_2_O_3_ lattice oxygen species. As suggested from the XRD results above, in the as-prepared fresh sample, carbonate is highly dispersed in La(OH)_3_ phase. The strong interaction between carbonate and hydroxide or formed complex compounds does have an effect on the electronic structure.

In the end, the CO_3_^2−^ group is the only species with consistent O 1s binding energy in Figure 3B. Keeping the hydroxyl peak with fixed FWHM, fitting of the O 1s spectrum taken at 200°C results in a carbonate peak at 532.2 eV and hydroxyl peak at 531.4 eV. At 300°C, the hydroxyl peak disappears and the O 1s spectrum can be well fitted with two peaks. After heating to 400°C, the carbonate O 1s peak relative intensity continuously decreases, which agrees well with the increase of the carbonate (O4c) species discussed above. At 400°C, the carbonate-related O 1s peak is observed at 532.3 eV, which is initially (in the as-prepared sample) a small shoulder at the same position.

The behavior of the other C 1s main peak at 284.6 eV is rather elusive for a simple explanation comparing with the other features. It is generally attributed to adventitious carbon accumulated on a sample surface before XPS analysis. In this section, only an objective description is provided and a tentative assignment is provided at the end on the basis of all results. Binding energy of this peak increases to 285.2 eV (200°C) and 285.5 eV (300 and 400°C). The atomic percentage of this peak carbon increases from 14 to 20% as the sample heated from 200 to 400°C (Supplementary Table S1). The assignment of this peak will be provided at the end of the whole *in situ* heating process description.

Combining the XPS and XRD results, it clearly suggests that the as-prepared fresh sample, although to be synthesized as La(OH)_3_, is rather easy to absorb CO_2_ from air (during synthesis and preparation on XPS sample holder) forming highly dispersed carbonate compounds in the bulk. If the sample is kept at temperature lower than 200°C, La(OH)_3_ still dominates in the bulk structure, and especially in the near surface region.

#### *In Situ* Heating From 500 to 800°C

Taking into account previous literature studies (Chen et al., 2014), TGA and online MS results, there are 3 steps in which the adsorbed species desorb or decompose during the heating (in the range 25–800°C). As the sample temperature continues to approach 600°C, *in situ* XRD obtained at this step demonstrates the patterns similar to that obtained at 300 and 400°C. The main difference is that the peaks become sharper than in the patterns collected at lower temperatures indicating the polycrystalline grain size is increasing. Following the 1^st^ step (25–400°C) data presented in Figure 3, the surface electrical structure transformation during the 2^nd^ step (400–600°C) is investigated. Increasing temperature to 500°C gives rise to further decrease of carbonate feature at 290.4 eV in the C 1s spectrum. Meanwhile, in the O 1s spectrum, the intensity of carbonate component at 532.5 eV changes from slightly higher than the lattice oxygen peak to significantly lower.

Carbonate thermal decomposition of bulk La_2_O_2_CO_3_ through online MS-measured TPD has been studied in our recent publication (Guan et al., 2021). It was concluded that the desorbed CO_2_ within temperature range from 400 to 600°C is mostly related to carbonates located on the surface and subsurface sites. As the result, the CO_2_ loss observed by TGA and online MS in Figure 2 does not significantly affect the XRD pattern as this step does not include decomposition of the bulk components. On the other hand, XPS, as a surface sensitive method, indicates that the surface carbonate species content gradually reduced by heating. Overall, the TGA/MS, *in situ* XRD, and XPS agree very well with each other confirming the CO_2_ removal in the 2^nd^ step is mostly related with surface and subsurface carbonates.

**FIGURE 4:**
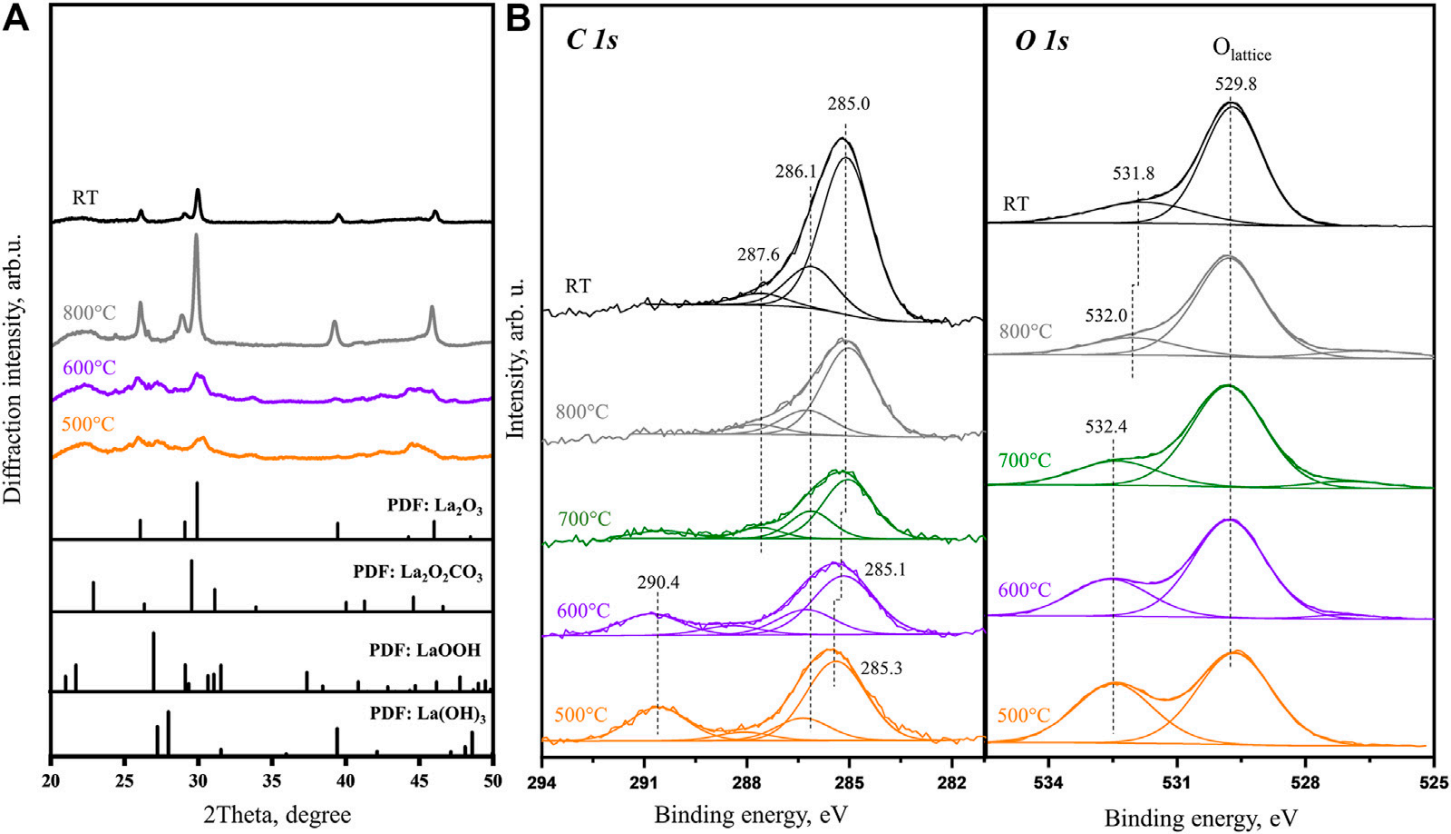
**(A)** *In situ* XRD patterns obtained for La_2_O_3_ sample at 500, 600, and 800°C with PDF database of references. **(B)** *In situ* XPS C 1s and O 1s core level spectra obtained at 500, 600, 700, and 800°C and after subsequent cooling to room temperature.

The online MS and TGA show that most of the CO_2_ desorption are depleted at the temperature of 700°C and higher. The XRD patterns (Figure 4A) obtained at 800°C agree well with the PDF file of La_2_O_3_, which confirms that most of the bulk carbonates are decomposed. The XPS data collected in the same temperature range also show no carbonate features in C 1s and O 1s spectra (Figure 4B). In the C 1s spectra, there is a main peak at 285.0 eV with shoulder peaks at 286.1 and 287.6 eV. According to the literature and our previous study results (Li et al., 2019), the main peak (at 285.0 eV) is attributed to the surface C–C feature, while the shoulder peaks are related to partially oxidized species, like C=O and C-O=C. On the other hand, in the O 1s spectra, the characteristic peak associated with the carbonate is gradually replaced by another new small shoulder, which is located at 532.0 eV at 800°C and at 531.8 eV after subsequently cooling to room temperature, while the main oxygen peak (529.8 eV) remains at the same position. Combined with our recent experimental results (Zhou et al., 2021), the small shoulder peak at 531.8 eV is assigned to the partially oxidized surface carbon species, which demonstrates the corresponding C 1s peak at 286.1 eV. According to the previous discussion, the main oxygen peak at 529.8 eV is the characteristic peak of the lattice oxygen in La_2_O_3_, which is the only La-containing species at this step. At temperatures higher than 400°C, when hydroxide is completely decomposed, the slow two-step carbonate decomposition process of first bulk carbonate (through the surface) and then surface carbonate can be easily correlated with *in situ* La_2_O_2_CO_3_ decomposition considered in Figure 1. The results indicate that only after heating to 800°C in an inert environment, the air exposed and stabilized La(OH)_3_ sample can be fully recovered to La_2_O_3_. This is also supported by the La 3d 5/2 spectra taken from room temperature to 800°C (Supplementary Figure S2), of which the peak splitting is increased from 3.8 to 4.6 eV, which also confirms that on the surface, carbonates and hydroxyl contamination is gradually removed and pure La_2_O_3_ is formed (Li et al., 2019).

**FIGURE 5:**
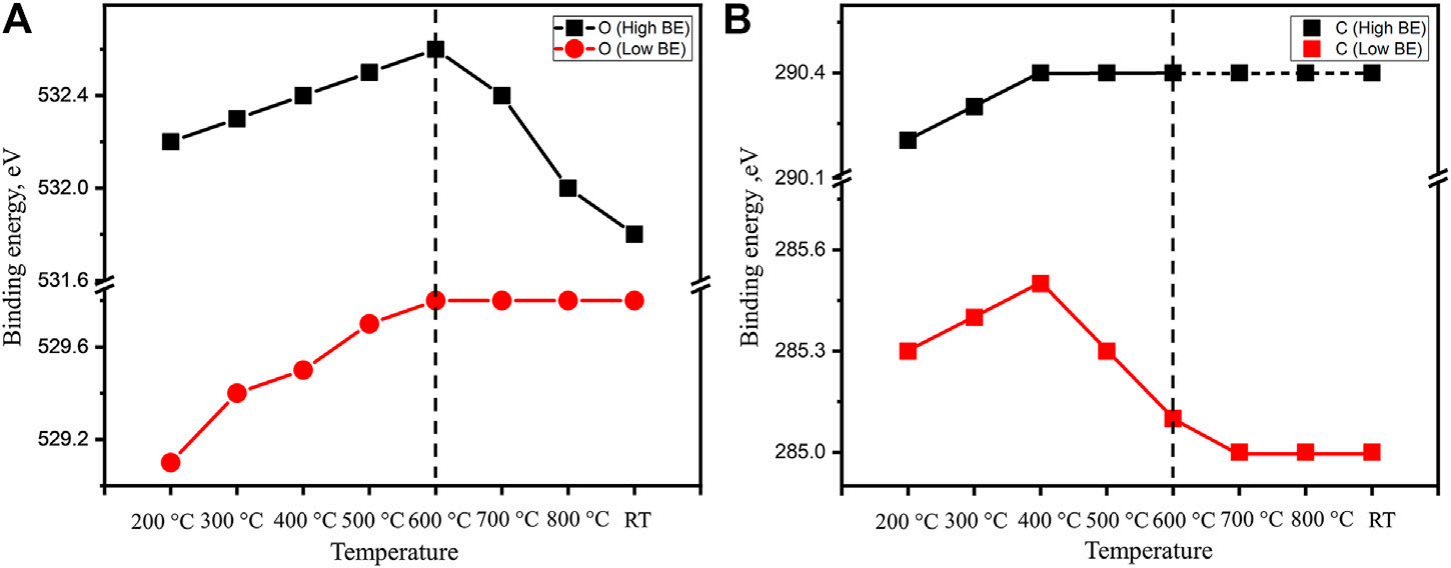
The binding energy evolution of main features in **(A)** O 1s and **(B)** C 1s of an as-prepared fresh La_2_O_3_ sample during the *in situ* hold at 200, 300, 400, 500, 600, 700, and 800°C and after following cooling to room temperature.

The evolution of both oxygen and carbon components depending on heating temperature is summarized in Figure 5. The C 1s binding energy of carbonate species is clearly approaching the previous calibrated characteristic values as discussed above. On the other hand, the lower binding energy peak in the C 1s spectra is always higher (285.0–285.5 eV) than the generally accepted value of adventitious carbon (284.8 eV). It is shown that the binding energy of this peak first goes up during the dehydration step and then decreases afterward. As the sample is heated to 700°C, this peak position is stabilized at a slightly higher position of 285.0 eV. Such a behavior of binding energy variation suggests that there is differential charging associated with carbon species. Unlike the bulk species of carbonates and oxide, the adventitious carbon is a surface species. During the heating, the sample volume is significantly reduced as the TGA above observes a relatively high mass loss of water and CO_2_ (molar ratio of H_2_O:CO_2_:La_2_O_3_ is roughly 3.4:1:2). The significant volume change can result in a loss of electrical contact between surface carbon species and the bulk of the sample which explains the charging built up during XP spectra collection.

## Conclusions

The temperature-correlated structure changes of two most popular La-related contamination species, *in situ*–prepared La_2_O_2_CO_3_ and *ex situ–*prepared La(OH)_3_, are investigated by *in situ* XPS, *in situ* XRD, and online MS. All observed species are further interpreted by previous obtained characteristic assignments and DFT surface structure model. After heated up to 800°C, both samples are completely regenerated to La_2_O_3_. For La_2_O_2_CO_3_ sample, during linear heating to 800°C, a partial carbonate coverage near the surface region is always observed, which confirms the previously suggested two-step model of bulk carbonate decomposition. The *ex situ–*prepared La(OH)_3_ sample is a hybrid of carbonates and hydroxyl due to air exposure–induced contamination. Three major steps of catalyst structure phase changes are investigated by the three *in situ* methods. Evolution of surface structures showing the changes of hybrid phases with hydroxyl and carbonates is observed by XPS and XRD from 200 to 800°C. Results provide surface and bulk structure details of the two samples during the heating treatment which bring insight into the activation and deactivation of La-containing materials.

## Data Availability

The raw data supporting the conclusions of this article will be made available by the authors, without undue reservation.
