# Supplementary material for: An In Situ Temperature-Dependent Study of La2O3 Reactivation Process
Source: Front Chem. 2021 May 31;9:694559. doi: 10.3389/fchem.2021.694559 (PMC8200846; doi:10.3389/fchem.2021.694559)
Supplement: Supplementary file 1 [file DataSheet1.DOCX]

Supplementary Material

An *in situ* temperature dependent study of La_2_O_3_ reactivation process

Xiaohong Zhou ^1,2,3^, Evgeny I. Vovk ^1^, Yang Liu ^1^, Cairu Guan ^1^, Yong Yang *^1^

^1^ School of Physical Science and Technology, ShanghaiTech University, 100 Haike Road, Shanghai, 201210, China

^2^ Shanghai Institute of Optics and Fine Mechanics, Chinese Academy of Sciences, Shanghai 201800, China.

^3^ University of Chinese Academy of Sciences, Beijing 100049, China

*** Correspondence:**Prof. Yong Yang
yangyong@shanghaitech.edu.cn

# Supplementary Figures and Tables

## Supplementary Figures


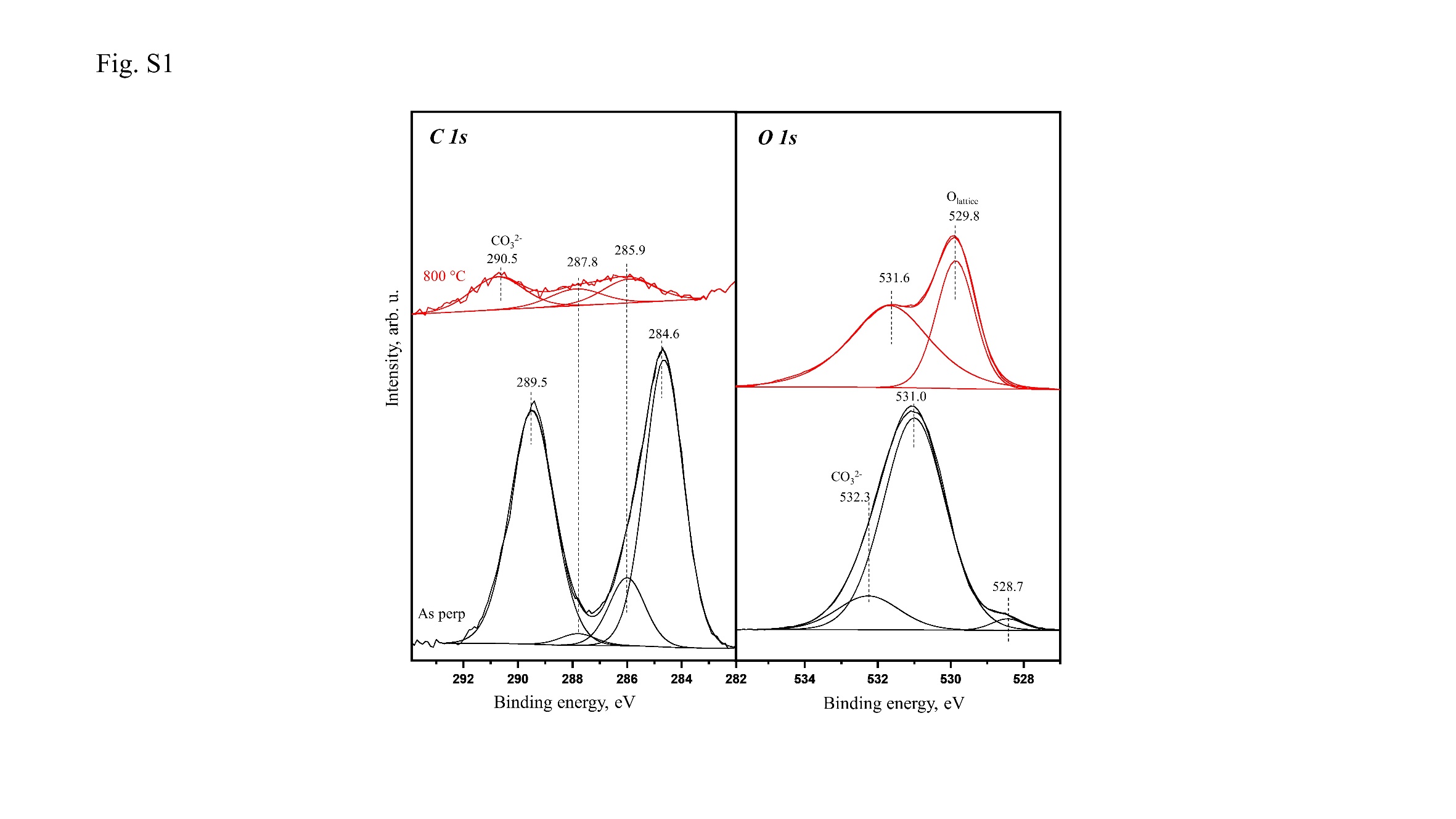


**Supplementary Figure1.** C 1s and O 1s core level XPS spectra of as prepared La_2_O_3_ sample (black), subsequent 10 min Ar^+^ etching (blue) and after heating in vacuum to 800ºC for 1 hour (red). All spectra are recorded at room temperature.


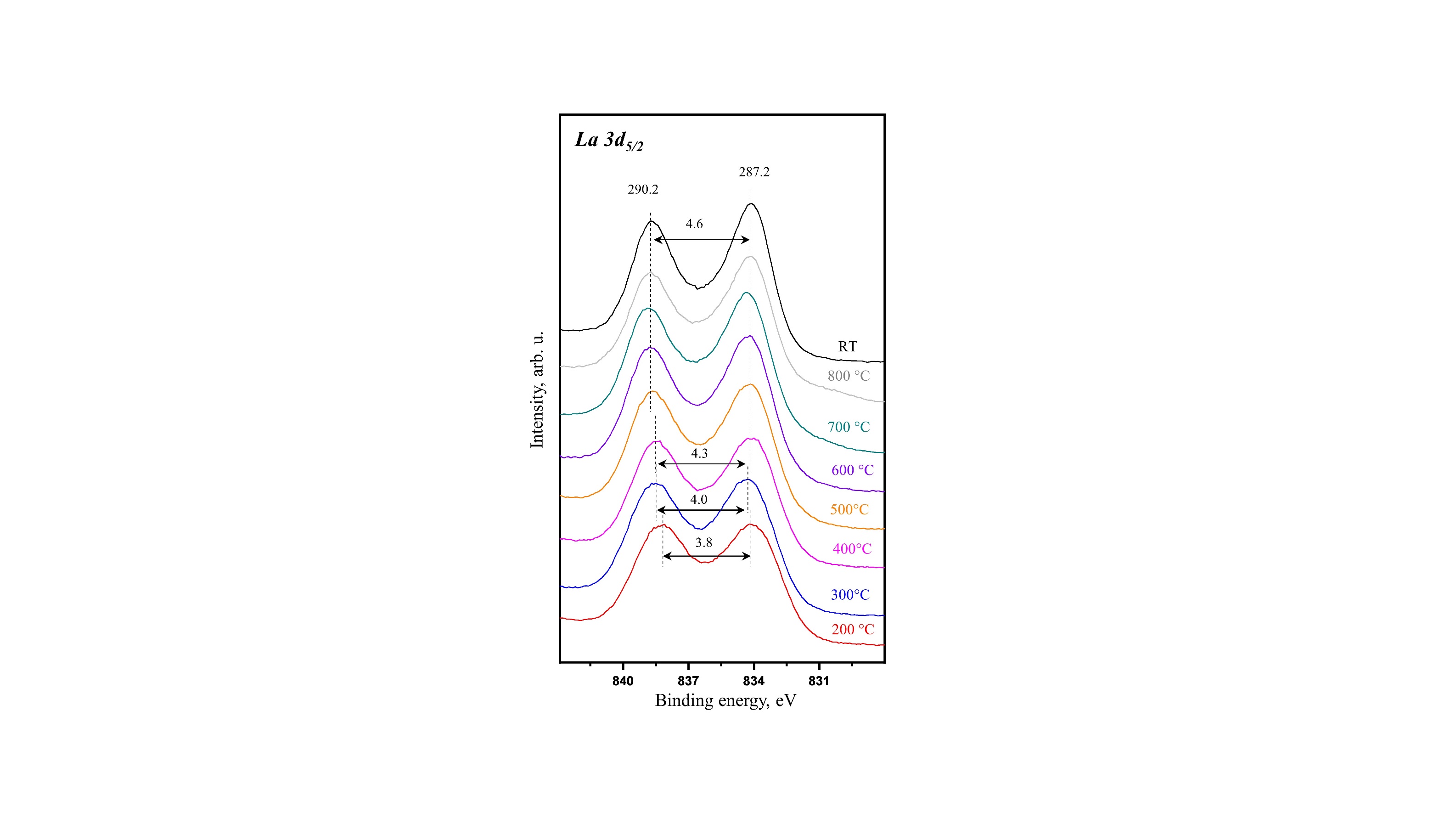


**Supplementary Figure 2.** *In situ* La 3d_5/2_ core level XPS spectra obtained for an as prepared fresh La_2_O_3_ sample and as the temperature is *in situ* hold at 200ºC, 300ºC, 400ºC, 500ºC, 600ºC, 700ºC, 800ºC, and followed by cooling down to room temperature in SAC.

## Supplementary Tables

**Supplementary Table 1.** Surface atomic concentration of oxygen, carbon and lanthanum in a fresh La_2_O_3_ sample under various heating temperature.

| Treatmens | Atomic concentration (%) | | | ΔE(La 3d_5/2_) |
| --- | --- | --- | --- | --- |
|  | O(tatal) | La | C(tatal) |  |
| as prep | 67.2 | 15.7 | 16.9 | 3.8 |
| 200°C | 52.4 | 24.6 | 23.0 | 4.0 |
| 300°C | 50.2 | 23.7 | 26.1 | 4.3 |
| 400°C | 48.6 | 25.5 | 25.9 | 4.4 |
| 500°C | 48.8 | 28.2 | 23.1 | 4.5 |
| 600°C | 47.8 | 32.4 | 19.8 | 4.6 |
| 700°C | 48.4 | 34.0 | 17.6 | 4.6 |
| 800°C | 47.2 | 32.4 | 20.4 | 4.6 |
| RT | 40.7 | 28.1 | 31.2 | 4.6 |
